# The Challenge of Triaging Chest Pain Patients: The Bernese University Hospital Experience

**DOI:** 10.1155/2012/975614

**Published:** 2011-10-26

**Authors:** Martin Rohacek, Amina Bertolotti, Nadine Grützmüller, Urs Simmen, Hans Marty, Heinz Zimmermann, Aristomenis Exadaktylos, Arampatzis Spyridon

**Affiliations:** ^1^Department of Emergency Medicine, University Hospital Bern, Freiburgstrasse, 3010 Bern, Switzerland; ^2^Statistical Consulting, Malzgase 9, 3042 Basel, Switzerland

## Abstract

Accurate diagnosis of the causes of chest pain and dyspnea remain challenging. In this preliminary observational study with a 5-year follow-up, we attempted to find a simplified approach to selecting patients with chest pain needing immediate care based on the initial evaluation in ED. During a 24-month period were randomly selected 301 patients and a conditional inference tree (CIT) was used as the basis of the prognostic rule. Common diagnoses were musculoskeletal chest pain (27%), ACS (19%) and panic attack (12%). Using variables of ACS symptoms we estimated the likelihood of ACS based on a CIT to be high at 91% (32), low at 4% (198) and intermediate at 20.5–40% in (71) patients. Coronary catheterization was performed within 24 hours in 91% of the patients with ACS. A culprit lesion was found in 79%. Follow-up (median 4.2 years) information was available for 70% of the patients. Of the 164 patients without ACS who were followed up, 5 were treated with revascularization for stable angina pectoris, 2 were treated with revascularization for myocardial infarction, and 25 died. Although a simple triage decision tree could theoretically help to efficient select patients needing immediate care we need also to be vigilant for those presenting with atypical symptoms.

## 1. Introduction

Chest pain is one of the most common causes for referral to emergency departments (ED), accounting for several million visits annually [1]. Acute disorders of cardiovascular and pulmonary function have significant morbidity, and the mortality of untreated acute myocardial infarction is up to 27% [2, 3]. All patients with chest pain and dyspnea—which may be symptoms of acute coronary syndrome (ACS)—are triaged with the highest priority in EDs, although only a small fraction of them require immediate care.

Recent updated NICE guidance recommends 12-lead electrocardiography (ECG) as soon as possible in patients with acute chest pain but warns not to exclude ACS if the ECG is normal [4]. The clinical assessments recommended to assess the likelihood of coronary heart disease (CHD) were studied in patients with stable CHD and are therefore not appropriate to assess the risk of ACS [5]. The TIMI risk score [6] was developed by a retrospective analysis of two phase 3 trials, the thrombolysis in myocardial infarction (TIMI) 11b trial [7] and the efficacy and safety of subcutaneous enoxaparin in unstable angina and non q-wave MI (ESSENCE) trial [8]. The TIMI risk score is a useful prognostic tool for patients with unstable angina pectoris and non-ST-segment elevation myocardial infarction (non-STEMI), but it has not been validated for the assessment of the risk of ACS in patients with chest pain or dyspnea presenting at the ED. A very recent meta-analysis did, however, show a linear relationship between TIMI risk score and short-term incidence of cardiac events in such patients [6]. We therefore conducted an observational study on clinician behavior and the likelihood of ACS in patients presenting to the ED with chest pain or dyspnea, with 5 years of followup. The aim was to establish how triage on admission might be optimized and to establish a decision tree that would help us to estimate the likelihood of ACS on admission.

## 2. Methods

This was a prospective observational study. To prevent selection bias, which has been shown to influence hospital admissions for myocardial infarction [9], and to provide an unbiased evaluation by avoiding confounding factors, we randomly selected 60 days during a 24-month period (December 2005 through November 2007) with proportional weekend-days and working-days. Not included were patients who were directly referred to the coronary laboratory. The following referral characteristics were assessed: self-referral, referral by a doctor or paramedic with suspected ACS, or for other reasons. The following patient characteristics were assessed: age, sex, diabetes mellitus, arterial hypertension, dyslipidemia, cigarette smoking, CHD, other cardiovascular diseases, other heart diseases, familial history of CHD, chronic lung disease, thromboembolic disease, and cancer.

The following symptoms on presentation were assessed: oppressive retrosternal chest pain radiating to arms or neck, oppressive retrosternal chest pain without radiation, respiration-dependent chest pain, stabbing chest pain, burning chest pain, dyspnea, cough, expectoration, heartburn, and the time of start of symptoms. 

Definition of events: dyspnea was defined as the perception of an inability to breathe comfortably [11]. Oppressive retrosternal chest pain was defined as a painful feeling of retrosternal pressure, tightness, or heaviness. Nonspecific chest pain was defined as nonoppressive, nonradiating, nonstabbing, nonburning and non-respiration-dependent chest pain. The primary outcome measure was a diagnosis of ACS in the ED after an interview by a trained physician, physical examination, ECG, and laboratory examinations. Radiological and echocardiographic examinations might also have been performed.

ACS was defined as acute myocardial infarction or unstable angina pectoris. Acute myocardial infarction was defined as an increase in troponin T to >0.01 ug/L, either initially or 6 hours later, with at least one of the following: ischaemic symptoms (chest pain, dyspnea), ECG changes indicative of ischaemia (ST-segment elevation or depression, or new left bundle-branch block), development of pathological Q waves in the ECG, imaging evidence of new loss of viable myocardium, or a new regional wall motion abnormality. ST-segment depression was defined as new horizontal or downsloping ST-segment depression ≥0.05 mV in two contiguous leads and/or T inversion ≥0.1 mV in two contiguous leads with a prominent R wave or R/S ratio >1. ST-segment elevation myocardial infarction (STEMI) was defined as an acute myocardial infarction with new ST-segment elevation at the J point in two contiguous leads with the cut-off points: ≥0.2 mV in men or ≥0.15 mV in women in leads V2 and V3 and/or ≥0.1 mV in other leads [12].

Other defined differential diagnoses were musculoskeletal chest pain (consistently reproducible chest wall tenderness or pain) and panic attack, defined according to the Diagnostic and Statistical Manual of Mental Disorders (DSM-IV-PC). Results of coronary catheterization were collected from medical records. Followup was performed in April 2010 by contacting the patients by phone. Secondary outcome measures were coronary catheterization, revascularization, myocardial infarction, and death before followup. The study protocol was approved by the local ethics committee. Patients signed an informed consent form.

## 3. Statistical Analysis

Separate logistic regression models for each variable provided odds ratios, 95% CIs, and the corresponding *P* value for the given groups of the outcome variable. Additional models were performed to adjust for age and gender. Conditional inference trees were used to estimate a regression relationship by binary recursive partitioning in a conditional inference framework [13]. *P* value was significant if <0.05.

All analyses were performed using R version 2.9.2 (R Development Core Team (2009). R: A language and environment for statistical computing. R Foundation for Statistical Computing, Vienna, Austria. ISBN 3-900051-07-0, available from: http://www.R-project.org.

## 4. Results

From December 2005 through November 2007 on 60 randomly selected days, 301 patients presented at our ED with chest pain or dyspnea. Patient characteristics are shown in Table 1. The median age was 56 years (range 17–92); 190 (63%) were male. 204 (68%) patients were referred with chest pain of unknown origin, 71 (24%) with dyspnea of unknown origin, 32 (11%) with suspected ACS, and 9 (3%) with suspected pulmonary embolism. 130 (43%) patients referred themselves, 134 (45%) were referred by a physician, and 35 (12%) by ambulance personnel. 234 (78%) reported chest pain, 74 (25%) dyspnea. Symptoms started up to 6 hours before entering the ED in 82 (27%), 6 hours to 1 day in 73 (24%), and more than one day in 126 (42%) patients; this information was not available for 20 patients.

Troponin T was measured in 235 (78%) patients. Troponin was not determined if the cause of chest pain could be attributed to noncardiac symptoms based on the clinical findings of the attending ED physician. In 42 (14%) patients, troponin T was measured a second time 6 hours later, because the chest pain lasted less than 6 hours. An ECG was performed in 279 (93%) patients. If the cause of chest pain could be determined by a method other than ECG (e.g., pleuritic chest pain caused by pneumonia diagnosed by chest X-ray), an ECG was not performed. The final diagnoses in the ER are shown in Table 2. Most common were musculoskeletal chest pain (27%), ACS (19%), and panic attacks (12%).

Out of 56 patients with ACS (16 with STEMI, 28 with non-STEMI), 23 had signs of myocardial ischaemia in the ECG. Coronary catheterization with or without PCI was performed within 24 hours in 51 (91%) patients with ACS. A culprit lesion explaining ischaemic symptoms was found in 44 (79%) patients. Vasospasm was found in 1 patient, 3 patients showed normal coronary arteries, and 3 patients showed good results after earlier PCI. Of the 5 patients without coronary catheterization, one had non-STEMI because of cocaine abuse, one died because of STEMI and cardiogenic shock, one with a non-STEMI received conservative management, one with unstable angina pectoris was sent home for an outpatient stress test, and one patient with non-STEMI did not undergo PCI for unknown reasons.

The results of the logistic regression models are outlined in Table 3. After adjusting for age and gender, the following variables were significantly associated with ACS: referral with suspected ACS (OR 9.66, 95% CI 4.22–22.14, *P* ≤ 0.001); referral with dyspnea (OR 0.17, 95% CI 0.06–0.47, *P* = 0.001); other heart disease (OR 0.15, 95% CI 0.05–0.46, *P* = 0.001); symptoms lasting for one day or more (OR 0.22, 95% CI 0.94–0.52, *P* = 0.001); oppressive retrosternal chest pain radiating to the arms or neck (OR 40.09, 95% CI 16.15–99.50, *P* ≤ 0.001); chest pain (OR 4.84, 95% CI 1.78–13.17, *P* = 0.002); respiration-dependent chest pain (OR 0.18, 95% CI 0.04–0.77, *P* = 0.02); nonspecific chest pain (OR 0.12, 95% CI 0.035–0.39, *P* = 0.001); cough (OR 0.14, 95% CI 0.04–0.46, *P* = 0.001).

**Figure 1:**
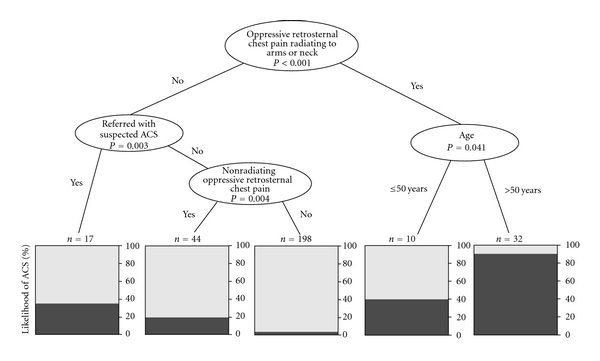
Conditional inference tree based on all clinical relevant variables associated with ACS: The boxes show the likelihood of ACS to be high (91%, *n* = 32), low (4%, *n* = 198), or intermediate (40%, *n* = 10, 35%, *n* = 17, and 20.5%, *n* = 44).

Figure 1 shows a conditional inference tree considering four variables associated with ACS that estimated the likelihood of ACS to be high at 91% in 32 patients, low at 4% in 198 patients, and intermediate at 20.5–40% in 71 patients.

Median followup time was 4.2 years (2.5–4.4). Followup information was available for 210 patients (70%).

Followup was available for 46 (82%) patients diagnosed with ACS: 33 had stable CHD, three underwent revascularization, two suffered myocardial infarction, and 8 died. 

Followup was available for 164 (67%) patients not diagnosed with ACS: 125 developed no CHD, 6 had coronary catheterization showing normal coronary arteries, one had coronary catheterization showing good results after earlier stenting, 7 underwent revascularization, 5 because of stable angina pectoris and 2 because of myocardial infarction, and 25 died. Information concerning the exact cause of death (e.g., cardiogenic or noncardiogenic causes) was not available. Median time between discharge from the ED to revascularization was 1.1 years (1 day–3 years).

Followup was available for 59 (72%) patients diagnosed with musculoskeletal chest pain: 51 patients developed no CHD, 4 underwent revascularization, one of whom had myocardial infarction, and 4 died.

Followup was available for 27 (71%) patients diagnosed with panic attack: none of the patients developed CHD, underwent revascularization, or suffered myocardial infarction, three underwent coronary catheterization showing normal coronary arteries, and one died.

## 5. Discussion

In this study in patients admitted to the ED with chest pain or dyspnea, the largest group of patients (27%) had musculoskeletal pain, 19% were diagnosed with ACS before leaving the ED, and 12% with panic attack. These findings are similar to earlier reports [14, 15]. Besides age, other cardiovascular risk factors were not significantly associated with ACS [16]. The same applied to dyspnea, also similar to earlier reports [10].

A further interesting finding of our study is that 43% of patients were self-admissions to the ED with chest pain or dyspnea. Statutory health insurance in Switzerland covers visits to any emergency healthcare facility which means that patients are free to seek medical advice without being referred by a primary care physician. Comparisons with other parts of Europe are therefore difficult, because the emergency services in Switzerland are often used instead of physicians in office practice, especially in towns and cities at night and during the weekend, which increases the burden on EDs at these times. This healthcare system-specific consultation pattern is a typical finding in Switzerland. For example in a recent analysis of a stratified sample of 1173 patients among 11258 ED admissions in our surgical emergency department concerning referral practice among Swiss and non-Swiss walk-in patients we found that nationality was associated with greater use of ED services for nonurgent problems. From all Swiss patients 67% referred themselves (walk-in patients) without a previous GP visit, compared to 79% self-referrals among foreigners. 83% of Swiss patients visited a GP regularly, compared with 57% of non-Swiss patients (498/598 Swiss versus 331/575 non-Swiss, *P* < 0.0001). Swiss citizens were also significantly more often admitted by their GP than non-Swiss patients. We believe that clinical and policy efforts must address barriers to GP care, since in the long term the GP provides better and more cost-effective care for patients with minor complaints [17].

In addition, 42% of our patients had had chest pain for >24 hours. Some of the patients in our catchment area live in remote valleys or in mountainous areas, up to 1900 meters above sea level, which means that some of them cannot consult a doctor immediately. The severity of the symptoms on presentation might also have played a role in the referral time point. A further explanation may be the specialty and experience level of the referring physician and the selection bias in referral rates of men and women with suspected angina, since angina pectoris symptoms tend to be more subtle in women than in men [18].

Based on the present study, after analysis of variables significantly associated with ACS, we selected four quickly assessable variables which can be used to create a conditional inference tree (Figure 1). The practical value of such an evaluation is to estimate the likelihood of ACS as high, intermediate, or low and to assist in the triage of high-priority patients in EDs [10, 19, 20].

In our study, 91% of the patients with ACS underwent coronary catheterization within 24 hours with identification of a culprit lesion confirming the diagnosis in 79% of cases. In other studies designed to establish clinical rules to predict the presence of ACS, coronary catheterization was either performed in only 19% of patients with ACS [21] or was used only as part of a combined outcome measure during followup of 30 days or two years, or its role was not described [10, 20, 22–27]. 

Based on our own findings, we feel that it would be interesting to set up a prospective study to validate this triage system and investigate the extent to which overtriaging might be reduced, together with patient outcome. Since some of our non-ACS patients later underwent coronary catheterization, after triaging patients on presentation to the ED, further assessment is necessary based on clinical skills and other risk scores and guidelines for the management of patients with ACS [4, 28, 29]. Also, improvement of algorithm parameters could be validated, for example, by targeting selected ACS and non-ACS patients. Early identification of ACS symptoms with a more structured and better organized followup together with an increased awareness level on the part of referring GPs might improve long term outcome in patients.

Our study had some limitations: firstly, troponin T, an important element in the definition of the primary outcome variable ACS, was measured only in 78% of patients. Secondly, 30% of patients were lost to followup because early follow-up was not performed. Thirdly, our sample size was small and represents experience at a single institution in a tertiary care ED setting and may therefore not be generalizable to other populations. Only 37% of our patients were women. Women with myocardial ischaemia can present with atypical symptoms [30]. Furthermore, we did not determine the cause of death in the follow-up cohort. Finally, despite randomization, some bias may have been introduced.

## 6. Conclusion

Estimation of the incidence of ACS and other cardiovascular events in emergency medicine is a dynamic field, and the accurate diagnosis of the causes of chest pain and dyspnea remains challenging. In this preliminary observational study, we attempted to find a simplified approach to selecting patients needing immediate care based on the initial evaluation of the ED physician and patient-derived information. Although a simple triage decision tree could theoretically help to select patients needing immediate care and thus further enhance the quality of care and optimize the allocation of limited resources in ED, since out of 91% patients primarily diagnosed with ACS 79% did have lesions at coronary catheterization; a minority of non-ACS patients also underwent coronary catheterization at a later timepoint after ED presentation. This suggests that despite using decision trees in the ED based on screening rules, we not only need to be vigilant for those presenting with typical symptoms, but also need to use our clinical skills and experience to identify and follow up patients who appear primarily not to have ACS.

## Figures and Tables

**Table 1:** Characteristics of 301 Patients.

Age, years, median (range)	56 (17–92)
	*n* = (%)
Male/female	190 (63%)/111 (37%)
Coronary heart disease	64 (21%)
Other cardiovascular disease	32 (10%)
Other heart disease	57 (19%)
Chronic lung disease	38 (13%)
Diabetes mellitus	39 (13%)
Arterial hypertension	119 (40%)
Cigarette smoker/history of smoking	129 (43%)
Dyslipidemia	36 (12%)
Familial history of coronary heart disease	49 (16%)
History of thromboembolic disease	18 (6%)
Cancer	22 (7%)
Chest pain	234 (78%)
Oppressive chest pain radiating to arms or neck	42 (14%)
Nonradiating oppressive chest pain	50 (17%)
Respiration-dependent chest pain	55 (18%)
Stabbing chest pain	55 (18%)
Nonspecific chest pain	89 (30%)
Burning chest pain	8 (3%)
Dyspnoea	74 (25%)
Cough	67 (22%)
Expectoration	19 (6%)
Heartburn	14 (5%)

**Table 2:** Final diagnosis at the ED of 301 Patients^†^.

	*n* = (%)
Musculoskeletal chest pain	82 (27%)
Acute coronary syndrome	56 (19%)
ST-elevation myocardial infarction	16 (5%)
Non-ST-elevation myocardial infarction	28 (9%)
Unstable angina pectoris	12 (4%)
Panic attack	38 (12%)
Cardiac arrhythmia	31 (10%)
Chronic lung disease	22 (7%)
Heart failure	21 (7%)
Lower respiratory tract infection	16 (5%)
Dyspepsia	15 (5%)
Pulmonary embolism	10 (3%)
Hypertensive crisis	7 (2%)
Upper respiratory tract infection	6 (2%)
Vasovagal Syncope	5 (2%)
Pericarditis	3 (1%)
Abdominal disease	3 (1%)
Pneumothorax	1 (0.5%)
Other ^‡^	5 (2%)

^†^More than one diagnosis per patient possible.

^‡^Other: Newly diagnosed cancer, haematoma in the pouch of an implantable cardioverter/defibrillator, instent stenosis of the subclavian artery, skin infection, cerebral haemorrhage.

**Table 3:** Logistic regression models for each variable separately on ACS.

Variables	Odds ratio	95% CI	*P* value
Age	2	1.29–3.09	0.002
Gender (female : male)	0.4	0.20–0.80	0.0094
Coronary heart disease	2.82	1.50–5.31	0.0013
Other heart disease	0.29	0.10–0.83	0.021
Other cardiovascular disease	1.54	0.65–3.62	0.328
History of thromboembolic disease	0.87	0.24–3.11	0.828
Chronic lung disease	0.63	0.23–1.70	0.36
Diabetes mellitus	1.51	0.57–4.01	0.404
Arterial hypertension	0.68	0.34–1.37	0.281
Cigarette smoker	0.76	0.32–1.79	0.522
History of cigarette smoking	0.86	0.36–2.04	0.728
Dyslipidemia	1.24	0.44–3.49	0.688
Familial history of coronary heart disease	0.73	0.27–1.98	0.537
Cancer	0.24	0.03–1.87	0.175
Referred with chest pain	0.68	0.37–1.24	0.212
Referred with dyspnoea	0.27	0.10–0.70	0.0069
Referred with suspected ACS	10.8	4.86–23.9	<0.001
Symptoms lasting <1 hour	0.87	0.10–7.62	0.902
Symptoms lasting 1–6 hours	0.67	0.29–1.58	0.362
Symptoms lasting >6 hours	2.03	0.96–4.29	0.063
Symptom lasting one day or more	0.26	0.11–0.60	0.0015
Symptom chest pain	3.46	1.32–9.05	0.012
Oppressive retrosternal chest pain radiating to arms or neck	37.6	16.0–88.2	<0.001
Nonradiating oppressive retrosternal chest pain	1.29	0.61–2.71	0.5
Burning chest pain	0.62	0.075–5.13	0.656
Respiration-dependent chest pain	0.13	0.03–0.57	0.0064
Stabbing chest pain	0.29	0.10–0.85	0.023
Nonspecific chest pain	0.11	0.032–0.35	0.0002
Symptom dyspnoea	0.91	0.46–1.81	0.792
Cough	0.16	0.05–0.53	0.0027
Heartburn	0.72	0.16–3.31	0.672

## Appendix

Conditional inference tree analysis was applied to elicit possible combinations of two or more risk factors. A tree based model is a good exploratory tool to approximate a complex model. The advantages are clearness of interpretation and visualisation of complex interactions which are not covered by regression modelling. The disadvantage is the possible instability using strong correlated predictors and the splitting of continuous variables into classes. Details are described here:

- statistical learning from a regression perspective available from [31] http://en.wikipedia.org/wiki/Decision_tree_learning;
- original research on inference trees available from: http://statmath.wu.ac.at/~zeileis/papers/Hothorn+ Hornik+Zeileis-2006.pdf [32].
